# Multi-dimensional immunoprotection of *Ganoderma lucidum* spore oil in immunosuppressed mice via microbiome-proteome-metabolome network analysis

**DOI:** 10.1038/s41598-026-40137-x

**Published:** 2026-03-02

**Authors:** Shuqi Deng, Xiaoxiao Wu, Wendong Xu, Xu Wu, Hongfei Cai, Shengpeng Wang, Juyan Liu, Jiliang Cao

**Affiliations:** 1 College of Pharmacy, Shenzhen Technology University, Shenzhen, China; 2 Department of GCP Center, Ya’an People’s Hospital, Ya’an, China; 3 Cell Therapy & Cell Drugs Key Laboratory of Luzhou, Department of Pharmacology, School of Pharmacy, Southwest Medical University, Luzhou, China; 4 National Engineering Research Center of Pharmaceutical Processing Technology of Traditional Chinese Medicine and Drug Innovation, Guangzhou HanFang Pharmaceutical Co., Ltd, Guangzhou, China; 5 State Key Laboratory of Quality Research in Chinese Medicine, Institute of Chinese Medical Sciences, University of Macau, Macao, China

**Keywords:** *Ganoderma lucidum* spore oil, Immunity, Immunosuppression, Gut microbiota, Metabolomics, Proteomics, Biochemistry, Immunology, Microbiology

## Abstract

*Ganoderma lucidum* has a long-standing history of use as a medicinal mushroom, with its spore oil (GLSO) extracted from broken cell walls using CO_2_ supercritical extraction. However, there is a notable scarcity of experimental studies on the protective effects and underlying mechanisms of GLSO on immune function impairment. The present study aims to explore the characteristics that GLSO contributes to protecting immune functions in cyclophosphamide-induced immunocompromised mice through a multi-omics analysis approach. GLSO administration significantly improved serum hemolysin levels, macrophage phagocytosis, and NK cell activity in immunosuppressed mice. Metagenomics, metabolomic, and proteomic analyses revealed that the immune protection mediated by GLSO was associated with structural rearrangements within gut microflora and shifts in microbial diversity. Specifically, there was an increase in beneficial microorganisms and a decrease in pathogenic organisms, accompanied by various alterations in metabolites and protein expressions. The identified 5 metabolites (propionic acid, beta-glycyrrhetinic acid, 3-aminosalicylic acid, creatine, and 2-phenylacetamide) and 5 proteins (Slc9a9, Blm, Hk3, AP1M2, and J chain) might serve as potential mediators of GLSO to alleviate immune dysfunction collectively caused by CYP in immunosuppressed mice.

**Supplementary Information:**

The online version contains supplementary material available at 10.1038/s41598-026-40137-x.

## Introduction

*Ganoderma lucidum* ((Leyss. ex. Fr.) Karst (*G. lucidum*)), one of the prominent medicinal mushrooms in traditional folk medicine, has been utilized for centuries under several names—Lingzhi in China, Reishi in Japan, and Mannentake in Korea—each synonymous with health and wellness. Currently, cutting-edge research is uncovering the bioactive components of *G. lucidum* and their implications for human health. *G. lucidum* is predominantly consumed as wall-broken extracts, particularly spore powder, which has been shown to enhance immune cell activity significantly. Studies reveal that *G. lucidum* spore powder promotes the secretion of inflammatory factors by macrophages and stimulates interferon production, ultimately fortifying the body’s immune response^1–3^. Additionally, *Ganoderma lucidum* spore oil (GLSO), derived from spore powder through wall-breaking technology, has shown various biological activities in pharmacological studies, including immune function regulation, anti-tumor effects, anti-inflammatory properties, antioxidant capabilities, and anti-aging benefits^4–7^. Recent studies have shown that GLSO treatment enhances the immune response and limits tumor growth through the macroscopic regulation of immune organs, the enhancement of immune cell responses, and the regulation of cytokine secretion in H22 tumor-bearing mice^8^. Besides, GLSO may alleviate granulomatous pulmonary nodules by regulating PI3K-Akt-mTOR signaling pathway, inhibiting p-AKT and p-mTOR activation, correcting metabolic disorders, and reducing the release of inflammatory factors and chemokines^9^. While previous research has indicated that GLSO may exert a certain regulatory effect on immune function in healthy individuals^10^, there remains a crucial gap in understanding its role in protecting immune function for immunocompromised hosts.

Nowadays, deviations in gut microbiota are linked with various diseases, including obesity, type 2 diabetes, hepatic steatosis, inflammatory bowel diseases, and several forms of cancer^11^. The exploration of gut microbiota composition and function, as well as its role in human health, has become an important focus of research in recent years. The human gut microbiota is increasingly regarded as a significant pleiotropic regulator of the host’s immune system, as it influences the levels of secondary metabolites, gene expression, and intestinal barrier permeability^12,13^. One of the key ways in which gut microbiota interacts with the host is through the production of microbial metabolites. These metabolites have been identified as crucial in guiding immune maturation, maintaining immune balance, regulating host energy metabolism and gene expression, as well as preserving mucosal integrity^14^. Therefore, the immunomodulatory effects of GLSO on various aspects of immunity, including innate and adaptive immunity, were investigated in a thorough manner using immunocompromised mice models established by cyclophosphamide. Meanwhile, combined with gut microbiota, serum metabolomics, and thymus proteomics analysis, this study sheds light on the multi-omic characteristic changes and potential mechanisms of GLSO on the protection of immune function.

## Results and discussion

### GLSO improved the immune function in CYP-induced immunosuppressed mice

**Fig. 1:**
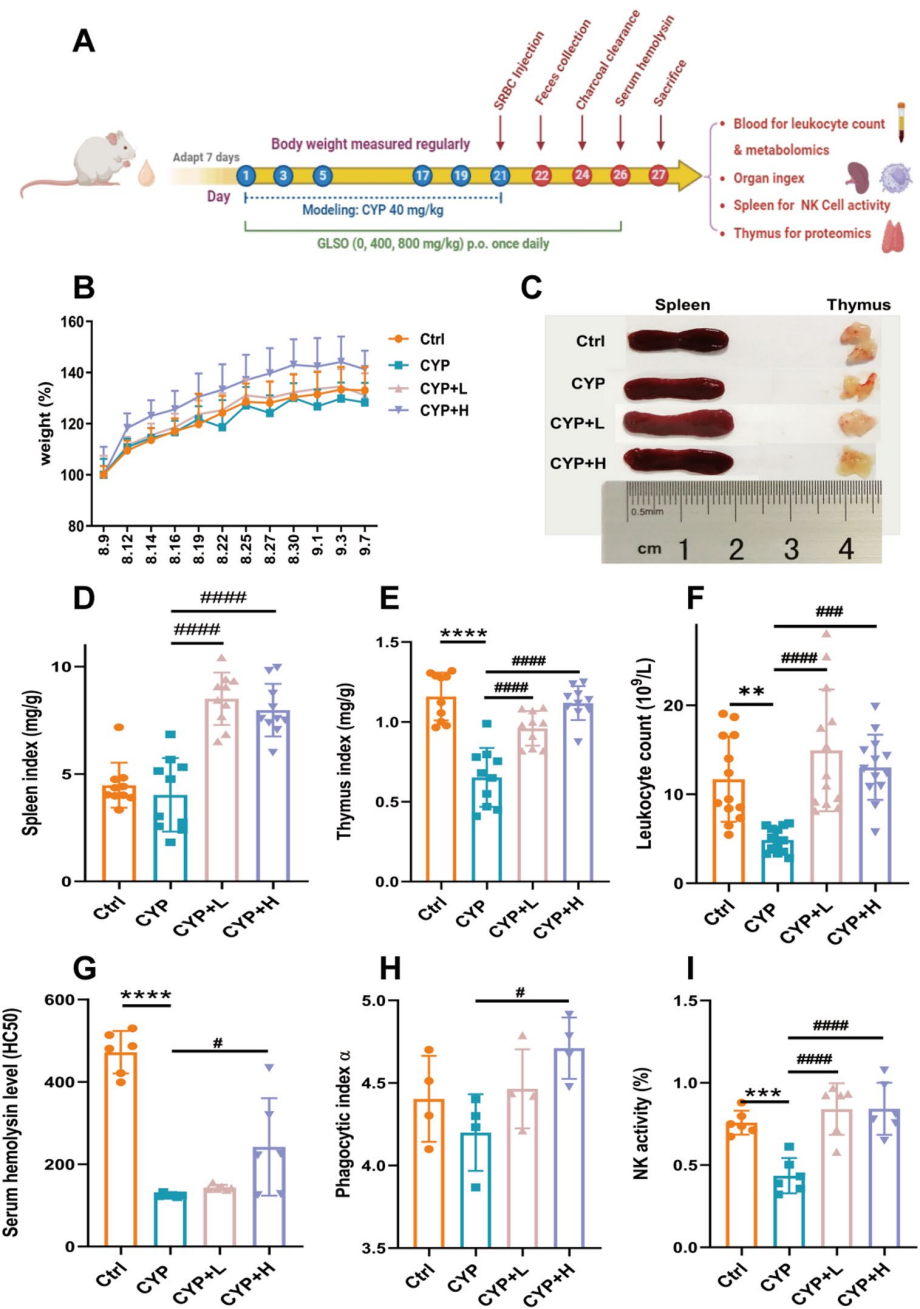
GLSO promoted immune protection in immunosuppressed mice induced by CYP. (**A**) Modeling, drug and administration, and sampling scheme. (**B**) Body weight gain of mice in Ctrl, CYP, GLSO_L, and GLSO_H groups (*n* = 26). (**C**) Morphology of spleen and thymus. (**D**) Spleen index (*n* = 10). (**E**) Thymus index (*n* = 10). (**F**) Leukocyte counts in mice blood (*n* = 13). (**G**) Serum hemolysin level (*n* = 6). (**H**) Macrophage phagocytosis is displayed by phagocytic index α (*n* = 4). (**I**) NK cell cytotoxicity (*n* = 6). Ctrl, normal control group; CYP, model group; CYP + L, model group + Ganoderma lucidum spore oil low dose group; CYP + H, model group + Ganoderma lucidum spore oil high dose group. Data are expressed as mean with SD. *p < 0.05, compared with Ctrl by one-way ANOVA followed by Dunnett post hoc test. ^#^*p* < 0.05, compared with CYP by one-way ANOVA followed by Dunnett post hoc test.

An investigation was conducted to examine the immune-enhancing effect of GLSO in immunocompromised mice, adhering to the experimental design in Fig. 1A. The dosages (400–800 mg/kg) of GLSO for mice were aligned with those recommended for humans. As a result, no statistically significant differences in body weight gain were observed between groups after modeling and GLSO administration (Fig. 1B). The morphological characteristics of the spleen remained stable across all groups (Fig. 1C); however, a significant reduction in both thymus size and indices was recorded in the model group (CYP) compared to the Ctrl group (Fig. 1E), thereby confirming the immunosuppressive effects of CYP. As presented in Figs. 1D, E, and F, the organ indices of the spleen and thymus, as well as the total leukocyte count in the blood, exhibited significant increases in the CYP + L and CYP + H groups compared to the CYP group, suggesting that GLSO could protect immune function in CYP-induced immunocompromised mice. To further assess the impact of GLSO on humoral immunity and cellular immunity, experiments involving serum hemolysis, carbon clearance, and natural killer (NK) cell activity were performed, with results illustrated in Figs. 1G, H, and I. The CYP group displayed a marked reduction in HC_50_ levels and NK cell activity compared to the Ctrl group, thus validating the success of the immunosuppression model established by CYP. Besides, the HC_50_ levels, phagocytosis index, and NK cell activity in the CYP + L and CYP + H groups were significantly higher than those in the CYP group, with statistical significance evident in the CYP + H group (*p* < 0.05). These findings suggested that high-dose GLSO was particularly effective in enhancing immune responses in immunocompromised mice. In conclusion, this study successfully established a model of immunosuppression using cyclophosphamide, demonstrating that GLSO could protect immune function to varying degrees, with a pronounced effect observed at higher doses.

Cyclophosphamide (CYP), an established immunosuppressant possessing anti-neoplastic properties, is widely utilized in clinical practice for treating malignant lymphomas and various bone marrow tumors, and others^15^. Nonetheless, CYP administration is associated with adverse effects, including disruption of DNA synthesis, inhibition of healthy cell proliferation, and attenuation of innate and adaptive immune responses^16^. At elevated dosages, CYP has been reported to compromise gastrointestinal mucosa, augment intestinal permeability, facilitate the translocation of pathogenic microorganisms, and provoke gastrointestinal distress^17,18^. The resulting myelosuppression and immunosuppression, along with its nonspecific cytotoxic properties, render it an effective agent for inducing immunodeficient animal models^12,19^. Besides, it has been found that GLSO can be used as a complementary treatment in chemotherapeutic anti-cancer strategies. The combination of GLSO and CYP effectively suppressed the lung metastasis of breast cancer by increasing the proportion of CD8 + Tcs in blood samples and enhancing the activity of NK cells in the spleen of mice, which enhance the immune activity of mice^20^. Therefore, a CYP-induced immunocompromised mouse model was established in this study to comprehensively investigate the effects of GLSO on immune protection. The above results confirmed the successful establishment of the immunocompromised mouse model induced by CYP, evidenced by a reduction in thymus organ index, serum hemolysin levels, and natural killer (NK) cell activity. Notably, treatment with GLSO, particularly at higher dosages, demonstrated a capacity to protect immune function, enhancing both innate and acquired immune responses by increases in spleen and thymus indices, leukocyte counts, serum hemolysin levels, phagocytic activity, and NK cell efficacy in immunocompromised mice.

### GLSO could alleviate the gut microbial disturbance induced by CYP

**Fig. 2:**
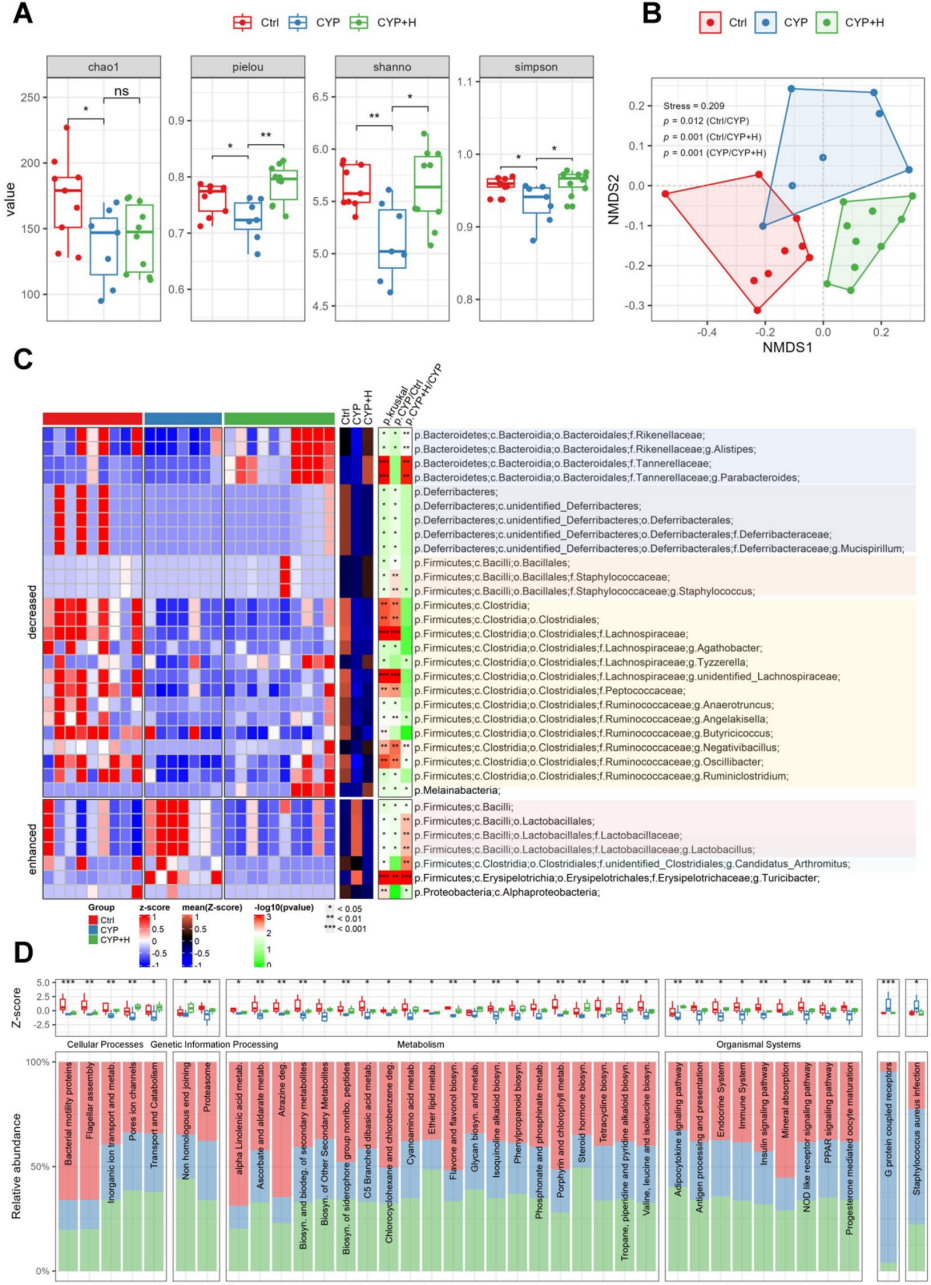
GLSO alleviated the gut microbial disturbance in immunosuppressed mice induced by CYP. (**A**) Microbial alpha-diversity was estimated by Chao, Pielou, Shannon, and Simpson indices in Ctrl (*n* = 9), CYP (*n* = 7), and CYP + H (*n* = 10) groups. Wilcoxon rank-sum test was used for the statistics. (**B**) An NMDS plot of bacterial communities and permutational multivariate analysis of variance were used to calculate significant changes between the three groups. (PERMANOVA p-value < 0.05). (**C**) Relative abundance of changed OTUs is illustrated by a heatmap (using R software (version 4.1.0, https://www.r-project.org/) with the ComplexHeatmap package (version 2.26.1; http://bioconductor.org/packages/release/bioc/html/ComplexHeatmap.html)). Red indicates high abundance and blue indicates low abundance. The Z-score method was used to calculate the normalized abundances for each OTU. The average Z-scores were colored blue and orange. Kruskal-Wallis test for three groups and Wilcoxon rank-sum test for two groups were used for the statistics to obtain p.kruskal and p.CYP/Ctrl or CYP + H/CYP. |Fold change| > 1.2. (**D**) Relative abundance of altered pathways in three groups based on KEGG database. Kruskal-Wallis test was used for the statistics (*p < 0.05, **p < 0.01, ***p < 0.001).

The gastrointestinal tract represents the largest immune organ in the human body, which plays a pivotal role in regulating immune homeostasis. Given the established link between GLSO-modulated intestinal microbiota remodeling and immune augmentation^21–23^, this research explored the alterations in gut microbiota associated with GLSO-mediated protection from immunosuppression. In prior experiments, the immune functional protection observed in immunocompromised mice was significantly more pronounced at elevated doses of GLSO; thus, this study focused on the high-dose group (800 mg/kg) to investigate the effects of GLSO on the intestinal microbial community. A total of 800 OTUs were identified from all fecal samples (Supplementary Table 1). Alpha diversity analysis was applied to evaluate community diversity across the groups. The results indicated (Fig. 2A) that Chao, Pielou, Shanno, and Simpson indices in the CYP group were significantly lower than those observed in the control group (Wilcoxon rank-sum test p-value < 0.05), while Pielou, Shanno, and Simpon indices showed marked enhancement in the CYP + H group. These findings indicated that immunocompromised mice were accompanied by a decrease in the diversity of their gut microbial communities and GLSO played a significant role in protecting the gut microbial diversity in immunosuppressed mice.

Furthermore, NMDS analysis was conducted based on the Bray-Curtis distance to investigate differences in species composition of intestinal flora among the Ctrl, CYP, and CYP + H groups. As illustrated in Fig. 2B, there are significant differences in the composition of the intestinal microbial community among the three groups, with community structures distinctly separated (PERMANOVA *p-*value < 0.05). This suggested that impaired immune function was associated with changes in the composition of the intestinal flora, while the treatment of GLSO resulted in structural remodeling of intestinal flora (PERMANOVA *p-*value < 0.05).

To find out the effective gut microbiota associated with the protection of immunity through GLSO treatment, the significant OTUs (Kruskal-Wallis test *p-*value < 0.05) were screened out at phylum, class, family, order, genus, and species levels. 7 immunodeficiency-enhanced OTUs (a mean intensity in the CYP group was higher than those in both the Ctrl and CYP + H groups, with a fold change > 1.2) and 26 decreased OTUs (Fig. 2C, Supplementary Table 1) were revealed. At the genus level, the presence of 10 gut microbiota taxa such as *Alistipes*,* Parabacteroides*,* Staphylococcus*,* Tyzzerella*,* Angelakisella*,* Negativibacillus*,* Oscillibacter*,* Lactobacillus*,* Candidatus_Arthromitus*, and *Turicibacter* achieved statistically significant protection with GLSO treatment compared to the CYP group, but 5 genera (*Mucisprillum*,* Agathobacter*,* unidentified_Lachnospiraceae*,* Anaerotruncus*,* and Ruminoclostridium*) remained down-regulated with treatment.

To further elucidate the biological functions affected by these gut microbiotas, we utilized the PICRUSt software to map read counts to pathways in the KEGG database. Similarly, these pathways with significant differences among the Ctrl, CYP, and CYP + H groups (Kruskal–Wallis test *p*-value < 0.05) were selected. There were 36 pathways attenuated and 2 pathways enhanced in the presence of impaired immune function (Fig. 2D, Supplementary Table 1). The pathways pertinent to organismal systems included the adipocytokine signaling pathway, insulin signaling pathway, PPAR signaling pathway, Antigen processing and presentation, NOD-like receptor signaling pathway, G protein-coupled receptors, etc. Additionally, metabolic pathways predominantly encompassed Biosynthesis of Secondary Metabolites, Lipid Metabolism, Amino Acid Metabolism, and Xenobiotics Biodegradation and Metabolism.

Research suggests that a decrease in both the abundance and diversity of gut microorganisms can disrupt the immune barrier of the gastrointestinal tract, increasing the host’s susceptibility to pathogenic microorganisms^24^. This dysregulation of intestinal flora has been implicated in immune dysfunction, precipitating the onset and progression of various diseases^25^. A critical immunomodulatory mechanism employed by gut microbes involves the production of short-chain fatty acids (SCFAs), predominantly butyric acid, which are synthesized by intestinal bacteria. These SCFAs play a vital role in promoting the development of regulatory T cells, reinforcing the mucosal barrier^26^. Notably, genera such as *Agathobacter*,* Unclassified_Lachnospiraceae*,* Anaerotruncus*,* and Ruminoclostridium*, the principal producers of short-chain fatty acid^27^, exhibited significant down-regulation in the CYP group. *Mucispirillum* resides within the mucus layer over colonocytes, contributing to the maintenance of mucosal homeostasis; its loss could lead to the compromise of the intestinal barrier^28^. Therefore, our results showed that the down-regulation of these five genera in response to CYP treatment could not be protected by GLSO, likely due to the degradation of the intestinal mucus layer combined with insufficient SCFA levels, which aggravated the increase of intestinal permeability and impairs intestinal immune barrier. Moreover, *Angelakisella* has been positively associated with butyrate^29^, which has various physiological effects, including the strength of intestinal barrier function and mucosal immunity^30^. The *Oscillibacter* and *Parabacteroides* are correlated with T-cell differentiation by promoting and sustaining the IL-10-producing Treg cells^31^. Proliferation of *Negativibacillus* has been linked to the development of intestinal villi for intestinal nutrient digestion and absorption^32^. Besides, *Tyzzerella* could abundantly produce aromatic amines which stimulate cytokine or immunoglobulin secretion in T or B cells^33,34^. It has been shown that *Alistipes* has protective effects in diseases such as colitis, autism spectrum disorder, and various hepatic and cardiovascular fibrosis^35^. Conversely, *Turicibacter* has been negatively associated with host immunocompetence, specifically antioxidant enzyme activities (e.g., CAT, SOD, GSH-Px) and pro-inflammatory cytokines (e.g., IL-1β, TNF-α), while positively correlated with the oxidative stress marker MDA^36^. Importantly, our experiment demonstrated that GLSO administration effectively reversed the microbial dysbiosis characterized by decreased beneficial bacteria and increased pathogenic taxa mentioned above in CYP-induced immunosuppressed murine models. However, *Lactobacillus* and *Candidatus_Arthromitus* were typically considered probiotics, while *Staphylococcus* acted as an opportunistic pathogen, maintaining a dynamic equilibrium that participates in immunoregulation. In this study, the observed decrease in Staphylococcus, alongside an increase in the abundances of *Lactobacillus* and *Candidatus_Arthromitus* of immunosuppressed mice, suggested that these latter genera were resilient to CYP-induced injury and their antagonistic bacterium decline contributed to their survival with richly nutrient utilization. In conclusion, GLSO could reconstruct microbiota dysbiosis where beneficial-pathogenic interactions were disrupted, thereby protecting the immunoregulatory network.

Low microbial richness also correlates with various metabolic parameters such as serum insulin, HOMA insulin resistance, free fatty acids, and triglyceride levels in the plasma^37^. Our findings indicated that the altered gut microbiota might be related to impede the involved metabolic pathways such as biosynthesis of Secondary Metabolites, Lipid Metabolism, Amino Acid Metabolism, and Xenobiotics Biodegradation and Metabolism, leading to disorders in nutrient metabolism and absorption and the accumulation of toxic substances in the host. SCFAs, the most extensively studied bacterial metabolites, also act as signaling molecules to bind to G protein-coupled receptors (GPCRs) expressed on immune cells and adipocytes, playing a major role in energy homeostasis^38^. Therefore, it could be posited that microbial metabolites following GLSO administration might interact with GPCRs expressed on diverse cell surfaces, mediating functional outcomes through the modulation of downstream signaling cascades such as the Adipocytokine signaling pathway, Insulin signaling pathway, PPAR signaling pathway, Antigen processing and presentation, and NOD-like receptor signaling pathway. Importantly, these signal pathways are intricately linked to regulating energy, metabolism, inflammatory responses, and cell differentiation.

In conclusion, immunodeficiency is closely related to alterations in microbial composition, accompanied by a reduction in the diversity of intestinal flora. GLSO treatment appeard to protect microbial diversity with a characteristic of an increase in beneficial bacteria and a decrease in pathogenic bacteria, leading to a significant reorganization of the gut microbiome composition and improving the host’s metabolic adaptability in CYP-induced immunosuppressed mice. Furthermore, these altered microbial populations primarily regulated the body’s immune function by mediating the metabolite pathways and organismal systems such as the Adipocytokine signaling pathway, Insulin signaling pathway, PPAR signaling pathway, Antigen processing and presentation, NOD-like receptor signaling pathway, and G protein-coupled receptors.

### Effects of GLSO on serum metabolic profiles in immunocompromised mice induced by CYP

**Fig. 3:**
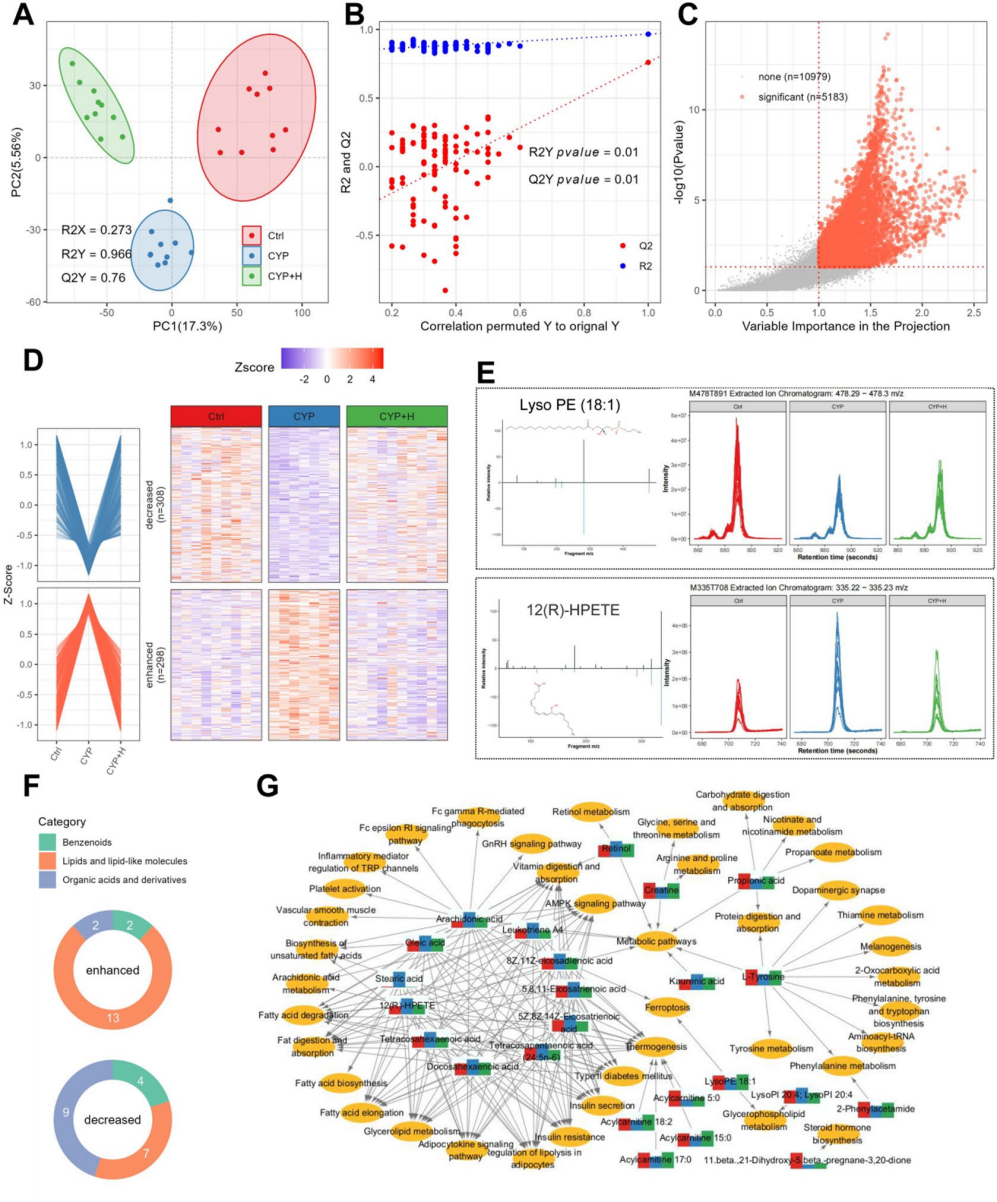
The dynamically changed serum metabolome in immunosuppressed and GLSO-treated mice. (**A**) PLSDA score plots of the three groups with different colors. The first two principal components of PC and PC were illustrated on the X-axis and Y-axis, respectively. (**B**) Cross-validation plot from 200 cycles permutation tests for performance evaluation of the PLSDA model. The blue and red dots represent the R^2^ and Q^2^ from the permutation test, respectively. (**C**) Scatter plot of VIP from PLSDA and *p*-value from Kruskal-Wallis test for significantly changed metabolite features. Two red dotted lines represent the cut-off s for VIP > 1 and p-value < 0.05. (**D**) A line plot (average intensity in each group) and a heatmap (intensity in each sample) (R software (version 4.1.0, https://www.r-project.org/) with the ComplexHeatmap package (version 2.26.1; http://bioconductor.org/packages/release/bioc/html/ComplexHeatmap.html)) were used to visualize significant changes in serum metabolite in immunocompromised mice. (**E**) MS/MS spectra and chromatogram extracted ion chromatograms (XIC) for example of immunocompromised enhanced and decreased metabolites. (**F**) Category count of identified differential metabolites. (**G**) Network of differential metabolites and the related pathways from KEGG database. (hypergeometric test p-value < 0.05).

There is a substantial interaction between gut microbiota and various complex metabolic axes in the organism. To evaluate the alterations in metabolite profiles caused by the changes in gut microbiota composition following GLSO treatment in immunocompromised mice, serum samples were used to perform a non-targeted metabolomics study based on UHPLC-MS to reveal the variable characteristics in the metabolic patterns mediated by GLSO in immunocompromised mice. From the non-targeted metabolomics analysis, a total of 12,277 quantified features in positive ion mode and 7,591 in negative ion mode were obtained (Supplementary Table 2). Differences in metabolites were detected through PLS-DA, and those metabolites with higher VIP scores (≥ 1.0) were further assessed using statistical tests (Kruskal-Wallis test, *p*-value ≤ 0.05) to determine the metabolites associated with immunodeficiency. As shown in Fig. 3A, the score plot exhibited a clear separation trend among the control, CYP, and CYP + H. Cross-validation was applied to confirm that the PLS-DA model did not suffer from overfitting, which was evidenced by the results of permutation test with *p*-values of R^2^ and Q^2^ both equal to 0.01 (Fig. 3B). From the analysis, 5183 metabolite features (Fig. 3C) were filtered to 298 immunodeficiency-enhanced features (mean intensity in the CYP group was higher than those both in Ctrl and CYP + H groups, fold change > 1.2) and 308 decreased features (Fig. 3D, Supplementary Table 2). By comparing similarity with the MS/MS spectral library (Fig. 3E), we identified 17 enhanced and 20 decreased high-confidence metabolites, predominantly comprising benzenoids, lipids, and organic acids (Fig. 3F, Supplementary Table 2). Utilizing the KEGG database, we constructed a network between altered metabolites and biological pathways (hypergeometric test *p*-value < 0.05) (Fig. 3G). This network revealed that the serum metabolites enhanced in immunodeficiency, especially lipids and lipid-like molecules such as arachidonic acid, oleic acid, stearic acid, 12(R)-HPETE (12R-hydroperoxy eicosatetraenoic acid), tetracosahexaenoic acid, docosahexaenoic acid, tetracosapentaenoic acid, eicosatrienoic acid, eicosadienoic acid, and leukotriene A4, were mainly participated in pathways related to fat digestion and absorption, biosynthesis of unsaturation fatty acids, arachidonic acid metabolism, glycerolipid metabolism, regulation of lipolysis in adipocytes, adipocytokine signaling pathways, insulin resistance, Type II diabetes mellitus, and AMPK signaling pathway, etc. Arachidonic acid was additionally implicated in inflammatory pathways, notably platelet activation, inflammatory mediators regulation of TRP channels, Fc epsilon RI signaling pathway, and Fc gamma R-mediated phagocytosis. Furthermore, the increased kaurenoic acid and Benzo(a)pyrene in the CYP group might be a potential immunosuppressive poison. Conversely, the metabolites that were decreased in immunodeficiency were mainly lipids and lipid-like molecules, as well as organic acids and derivatives, such as acylcarnitines, beta-Glycyrrhetinic acid, L-Tyrosine, creatine, propionic acid, Glu-Gln, Glu-Ile, 2-Phenylacetamide, Hexanoylglycine, Lyso PE, and 3-Aminosalicylic acid, etc.

Importantly, some metabolites have a direct link to the immune system. Findings from the microbiome analysis suggested that CYP-induced dysbiosis in gut microbiota might lead to a reduction of SCFA (mainly Butyric, acetic, and propionic acids), consistent with the decreased serum propionic acid levels observed in metabolomics analysis. It has been shown that propionate activates G protein-coupled receptors to increase adiponectin secretion, thereby promoting insulin release and sensitivity, as well as fatty acid oxidation^39^. In states of insulin resistance, the inhibitory effect of insulin on lipolysis is diminished, leading adipocytes to release elevated levels of fatty acids into the bloodstream. Metabolomics analysis revealed an increase in fatty acid concentrations (including Oleic acid, Stearic acid, Arachidonic acid, 5,8,11-Eicosatrienoic acid, 8Z,11Z-eicosadienoic acid, Docosahexaenoic acid, Tetracosahexaenoic acid, Tetracosapentaenoic acid (24:5n-6), 5Z,8Z,14Z-Eicosatrienoic acid) alongside a decline in acylcarnitine concentrations (notably Acylcarnitine 5:0, Acylcarnitine 15:0, Acylcarnitine 17:0, Acylcarnitine 18:2) in the CYP group. Acylcarnitines are critical for the transport of long-chain fatty acids into mitochondria for subsequent β-oxidation^40^. Consequently, the observed reduction in serum propionate levels in CYP-treated mice might disrupt adiponectin secretion within the adipokine signaling pathway, potentially aggravating the progression from insulin resistance to type 2 diabetes mellitus; the concomitant attenuation of AMPK and PPAR pathways further hampered fatty acid oxidation leading to fatty acid accumulation. These findings were highly consistent with the signaling pathways involved in the microbiome analysis. The current in vitro and in vivo researches consistently indicate that fatty acids at higher concentrations can suppress immune functions, particularly lymphocyte functions, and promote a shift in the Th cytokine balance toward a Th2-like profile, suggesting eicosanoid metabolites may inhibit and deviate from cellular immunological responses^41^. Besides, AA oxidative metabolites (such as arachidonic acid, 12(R)-HPETE, and leukotriene A4, as identified in this study) serve as potent mediators of the early (acute) inflammatory response, including vasodilatation, chemotaxis, neutrophil responses, and platelet aggregation^42^. Moreover, high intakes of long-chain n-3 PUFA (such as docosahexaenoic acid), suppress a wide range of immune variables, including lymphoproliferation, CD4 + cells, antigen presentation, adhesion molecule expression, as well as Th1 and Th2 responses^43^. Notably, the significantly elevated levels of kaurenoic acid in the CYP group have been reported to exhibit immunosuppressive effects by activating TGF-β signaling pathways while inhibiting NF-κB activity, resulting in increased levels of TGF-β and IL-10 cytokines and decreased production of IL-12^44,45^. Additionally, the heightened levels of Benzo(a)pyrene attenuated the aryl hydrocarbon receptor-induced differentiation of murine macrophages through aryl hydrocarbon receptor-dependent induction of IL-10^46^. In contrast, GLSO treatment significantly decreased the levels of the up-regulated aforementioned immunosuppressive metabolites in CYP-induced mice, protecting immune function in immunosuppressed mice.

Furthermore, GLSO administration also enhanced immune function through the elevation of beneficial metabolites (e.g., L-Tyrosine, Propionic acid, Acylcarnitine, Creatine, 2-phenylacetamide, beta-glycyrrhetinic acid, Hexanoylglycine, Lyso PE, Glu-Gln, Glu-Ile, and 3-Aminosalicylic acid). Notably, beta-Glycyrrhetinic acid not only significantly enhances specific antibody production and lymphocyte proliferation, increasing concentrations of IgG, and IgM, as well as the proportions of CD4 + and CD8 + T lymphocyte subpopulations by regulating humoral and cellular immunity, but also prevents free fatty acid-induced hepatic lipotoxicity via lysosomal and mitochondrial pathways^47,48^. Besides, 2-phenylacetamide has been identified to inhibit renal fibrosis via MAPK signaling pathway-mediated oxidative stress with reduced inflammation and cytokines in SHR Rats^49^. Obesity-associated glycine deficiency, with slow synthesis rates of several acyl glycines (such as Hexanoylglycine), impaired the body’s ability to eliminate endogenous and exogenous metabolites via the glycine conjugation pathway which was a phase II detoxification system in the liver^50^. Creatine is a vital nutrient that promotes macrophage function by increasing ATP levels^51–53^. L-tyrosine plays a pivotal role in the immune system by promoting cellular immune responses and regulating the proliferation and differentiation of immune cells^54,55^. Lyso PE (Lysophosphatidyl ethanolamine) can effectively promote the migration of killer T cells into the tumor, exhibiting anti-tumor properties^56^. Gut-restricted anti-inflammatory treatment with aminosalicylic acid not only prevents the intestinal inflammatory reaction associated with high-fat-diet-induced obesity but improves overall insulin resistance and alleviates low-grade inflammation in visceral adipose tissue^57^. Additionally, the GLSO treatment led to an increase in specific short amino acid motifs (Glu-Gln, Glu-Ile, etc.), which serve as substrates in protein and nucleic acid synthesis^58^.

Consequently, this might imply that the majority of metabolites that were altered by CYP and could be retained through GLSO treatment were involved in the regulation of immune responses, suggesting that the gut microbiome-host metabolic axis was critical for the effectiveness of GLSO on immune protection in immunocompromised mice. The accumulation of fatty acids and inflammatory toxins in CYP-induced immunosuppressed mice might be caused by insulin resistance and fatty acid synthesis which were mediated by the adipokine signaling pathway, while the AMPK signaling pathway was inhibited to block fatty acid β-oxidation. This ultimately led to inflammatory damage and immune imbalance. In contrast, GLSO could alleviate these detrimental metabolites, protecting the body’s immune function and enhancing overall health through related immune-stimulating components.

### GLSO-mediated alterations of the thymic proteome in immunocompromised mice induced by CYP

**Fig. 4:**
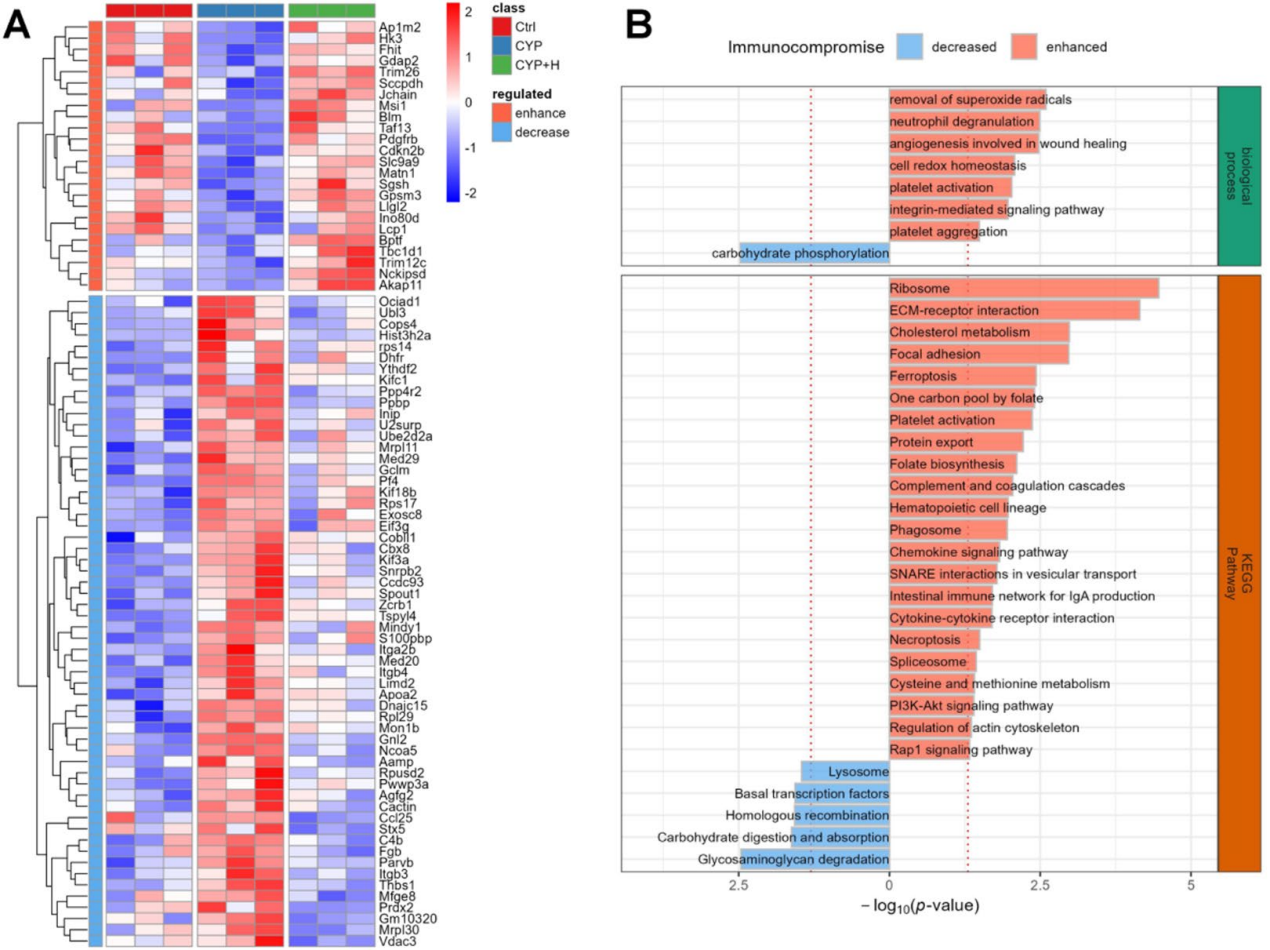
Differential thymic proteins in immunosuppressed and GLSO-treated mice. (**A**) Heatmap (using R software (version 4.1.0, https://www.r-project.org/) with the ComplexHeatmap package (version 2.26.1; http://bioconductor.org/packages/release/bioc/html/ComplexHeatmap.html)) for significant differential proteins in immunocompromised and GLSO-treated mics. Fold change > 1.2 and ANOVA p-value < 0.05. (**B**) Biological function analysis of differential proteins in GO and KEGG database. A hypergeometric test was used for enrichment statistics analysis. (Hypergeometric test p-value < 0.05)

The thymus, the small gland in the lymphatic system that makes and trains special white blood cells called T-cells, can reflect the body’s immune condition. To evaluate the effects of GLSO on protein expression levels in immunocompromised mics, the thymus samples were pooled and labeled one unit of TMT reagent, resulting in a 9-channel (3 biological replicates in each group) TMT-base proteome (Supplementary Table 3). Analyses revealed that 24 proteins exhibited significantly decreased expression in the immunocompromised state (ANOVA *p*-value < 0.05 and mean intensity in CYP was lower than those both in control and CYP + H groups, fold change > 1.2) and 58 proteins were enhanced (Fig. 4A). We found that immunocompromised enhanced proteins were involved in various biological functions (hypergeometric test p-value < 0.05), such as platelet activation (Itga2b, Itgb3, and Fgb), complement and coagulation cascades (C4b, Fgb), and the chemokine signaling pathway (Ppbp, Pf4, Ccl25) (Fig. 4B). Still, others regulated metabolism and intestinal barrier to influence immune function, such as Hist3h2a, Thbs1, Jchain, Mfge8, Slc9a9, Ap1m2, Blm, Apoa2, and Hk3.

It was noteworthy that the majority of differentially expressed proteins and signaling pathways identified in this study were associated with immune regulation within the body. CYP acts as an alkylating agent with strong immunosuppressive properties, mediating its cytotoxicity through DNA damage^59^. The p53 pathway activation promoted apoptosis, triggered by several stress signals such as DNA damage, oxidative stress, and activated oncogenes^60,61^. Specifically, CYP-induced DNA damage activated the p53 pathway, facilitating cellular apoptosis and the up-regulation of the THBS1 (a gene of thrombospondin-1 for angiogenesis inhibition and the promoting defective angiogenesis)^62–64^. Besides, CYP exposure could result in intestine oxidative damage with the characters of increased intestinal permeability, gastrointestinal mucosal barrier damage, intestinal flora disorder, and elevated risk of infections^65^, which was corroborated by microbiome analyses conducted in our study. Then, the initial damage induced by CYP evoked the complement system, a pivotal arm of innate immunity, to promote cascade reactions in response to pathogen-associated molecular patterns (such as pathogens), damage-associated molecular patterns (such as apoptotic cells), or structurally altered extracellular molecules^66^. However, excessive activation or insufficient regulation of this system on host cells can lead to an immune imbalance, potentially perpetuating a vicious cycle between complement activation, inflammatory cell recruitment, and tissue damage^67^. Notably, C4b (up-expressed complement component 4b gene in the CYP group) participates in the complement reaction by forming covalent linkages with antigen-antibody complexes or cellular surfaces^68^. In parallel, contact between blood and damaged blood vessels activated the coagulation cascade to promote the conversion of soluble fibrinogen (up-expressed Fgb gene in CYP group) to the insoluble fibrin clot and initiated platelet activation^69^. The integrin (up-expressed Itga2b, Itgb3 & Itgb4 genes in CYP) in activated platelets bound to fibrinogen (Fgb), triggering platelets adhesion, aggregation and spreading, additional granules secretion and clot retraction, ultimately leading to thrombus formation^70,71^. The key inflammatory function of platelets was the release of immunomodulatory mediators (also named granule mediators) such as chemokines, cytokines, and other mediators, which recruit and activate various immune cells (such as eosinophil, neutrophil, macrophage, T lymphocyte) or modulate endothelial cell activation to trigger acute inflammation^72^. The chemokines of Ppbp, Pf4, and Ccl25 (the up-expressed genes in CYP) participated in the chemokine signaling pathway to facilitate directional cell trafficking, regulate cellular differentiation, and influence apoptosis and ROS production^73^. Besides, activated platelets were shown to enhance leukotriene production from neutrophils via the transcellular metabolism of arachidonic acid, resulting in increasing vascular permeability and mucus hypersecretion, which can lead to edema^74–76^. This observation was aligned with the metabolomics analysis with elevated levels of leukotriene A4, arachidonic acid, and other eicosanoid metabolites in the CYP group. Simultaneously, activated platelets interacted with neutrophils to modulate their activation state, ultimately inducing the formation of neutrophil extracellular traps (NETs)^77^. The release of NETs inflicts severe damage to surrounding tissues, as a result of direct cytotoxicity on endothelial cells by histones^78^. The up-regulated histone H2A (Hist3h2a) in this study, as an attack site for alkylating DNA-damage agent (CYP) to induce chromatinolysis^79^, could be highlighted to involve in Necroptosis. In conclusion, the direct injury and the indirect-induced inflammatory response induced by CYP might lead to systemic inflammation-immune dysregulation, ultimately exacerbating thymic tissue damage with the up-regulation of identified genes. Conversely, GLSO might protected the host’s health by down-regulating these detrimental genes associated with disease progression.

The absence of specific genes expression in the CYP group was linked to metabolic disorders and tissue damage. Elevated levels of Mfge8 not only correlate with indicators of insulin resistance but also facilitate the absorption of dietary triglycerides and the cellular uptake of fatty acids^80,81^, which was consistent with the trends derived from metabolomics analysis. Deficiencies in macrophage clearance of apoptotic neutrophils might serve as a contributing factor to tissue damage, especially in the context of fatty acid accumulation such as oleic and palmitic acids^82^. Besides, the up-regulated APOA2 might be involved in fatty acid transport. When Slc9a9 is deficient, mainly expressed in human β-cells, this could lead to detrimental glucose tolerance with impaired insulin secretion^83^, thereby causing gut microbial dysbiosis and mucosal inflammatory response^84^. Moreover, a deficiency of AP1M2 has been associated with epithelial immune dysfunction with the characters of down-regulated antimicrobial proteins and impaired secretion of immunoglobulin A, which contributes to intestinal dysbiosis and heightened bacterial translocation within the mucosa^85^. In the absence of Blm, thymocyte populations are severely diminished, and T cells manifest defective homeostatic and TCR-induced proliferation, resulting in extensive chromosomal damage^86^. The lack of the J chain disrupts the transport of gut IgA, consequently undermining the development of intestinal antitoxic defenses^87^. CYP exposure has been shown to induce the macrophage polarization towards the M1 phenotype, which is directly associated with the inflammatory response, leading to multi-organ toxicity^88^. The HK3 gene, abundantly expressed in bone marrow and lymphoid tissues, facilitates the polarization of macrophages towards the M2 phenotype, which is a key process in anti-inflammatory response and tissue remodeling and repair^89,90^. Importantly, the dysregulation from the aforementioned gene expressions might be effectively alleviated after the GLSO administration.

Overall, the immune protection effect mediated by GLSO might be related to the down-regulation of pertinent immunosuppressive proteins and the up-regulation of immune-enhancing proteins.

### Correlation analysis explored the immunomodulatory influence of GLSO on the microbe-protein-metabolism axis in immunocompromised mice induced by CYP

**Fig. 5:**
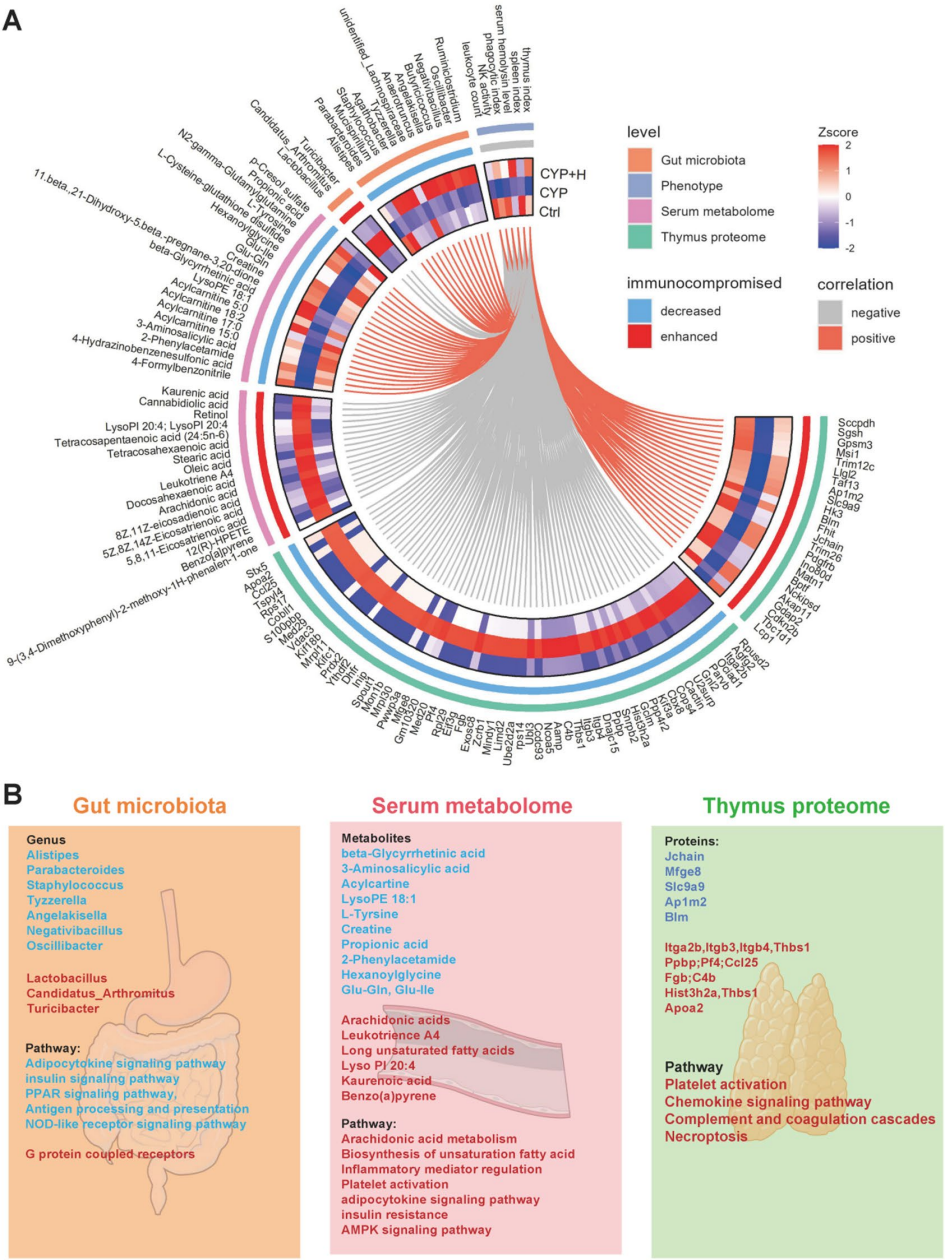
(**A**) Chord diagram about the correlation of the phenotypical measurement with microbiota genus, serum metabolites, and thymic proteins. (**B**) Cross-talk of key differential molecule and their pathways in three omics level.

Based on the above findings from immunofunctional evaluation, gut microbiota and thymus proteins, and serum metabolites, Spearman correlation analysis was performed to more intuitively illustrate the critical microorganisms, proteins, and metabolites that promote the protection of immunocompromised mice treated with GLSO (Fig. 5A). Serum hemolysin represents a specific antibody that reflects the proliferation and differentiation of hemolytic B cells^91,92^. The concentration of serum hemolysin in response to SRBC immunization serves as a direct indicator of humoral immune functionality. Moreover, macrophages act as specialized phagocytes within the innate immune system, exhibiting key processes such as antigen capture, endocytosis, and presentation. These functions establish macrophages as crucial intermediaries connecting innate and adaptive immune responses^93,94^. Consequently, the phagocytic activity of macrophages is frequently employed as a parameter for evaluating the nonspecific immune status in animal models^95^. NK cells play a pivotal role in the body’s defense against neoplasms and pathogenic infections. The assay of NK cell activity is instrumental in evaluating the impact of various substances on nonspecific cell-mediated immunity. Peripheral blood leukocytes constitute essential immune cells with diverse immunomodulatory roles. However, CYP has been associated with myelosuppression, exerting deleterious effects on hematopoietic and circulatory systems^96,97^. The depletion of hematopoietic stem cells and the resultant impaired capacity of bone marrow to generate new blood cells can lead to conditions such as leukopenia and oligocytosis, potentially culminating in severe morbidity and mortality^98^. Lymphoid organs, characterized by a predominance of lymphoid tissue, are responsible for generating lymphocytes and facilitating immune responses; thus, they are classified as immune organs, including the thymus and spleen. The indices of the thymus and spleen, which reflect lymphocyte proliferation, serve as estimators of the overall strength of immune function, albeit as more superficial and lagging indicators. According to the correlation network, the thymus index, spleen index, serum hemolysin levels, phagocytosis index, and NK cell activity were positively correlated with immunocompromised decreased molecules and negatively correlated with immunocompromised enhanced molecules.

The cross-talk of various molecules across different omics levels and their associated functions were meticulously constructed in this study. The potential features underlying GLSO-mediated protection of immune function in immunosuppressed mice are further elucidated in Fig. 5B. Our findings demonstrated that GLSO might protect innate and adaptive immunity by reestablishing the diversity and community structure of gut microbiota. Specifically, GLSO administration might rectify the CYP-induced gut bacterial imbalance, thereby promoting normal gut microbial metabolism to enhance nutrient absorption (Propionic acid, Creatine, etc.). Extensive research has evidenced that gut microbiota dysbiosis contributes to host insulin resistance, culminating in metabolic inflammatory responses associated with type 2 diabetes mellitus^99^. So, the insulin sensitivity pathway was likely attributed to the alteration of gut microbiota following GLSO administration, with increased metabolite of 3-Aminosalicylic acid and protein of Slc9a9 to alleviate insulin resistance. In addition, elevated propionate metabolites in serum might enhance fatty acid β-oxidation and metabolism by activating AMPK and PPAR signaling pathways, thereby mitigating metabolic inflammatory damage as a result of CYP-induced fatty acid accumulation. NK cell dysfunction due to insulin resistance and fatty acid accumulation could be restored^100^. Previous literature has reported that β-glycyrrhetinic acid exhibits immunomodulatory activity to inhibit human complement while simultaneously reducing the absorption of xenobiotics across small intestinal membranes through the up-regulation of efflux transporters^101,102^. We hypothesized that these five elevated metabolites (beta-glycyrrhetinic acid, 3-aminosalicylic acid, propionate, creatine, and 2-phenylacetamide) in serum and five proteins in thymus (Slc9a9, Blm, HK3, Ap1m2, and J chain) might serve as potential active substances to regulate immune protection after GLSO treatment, ultimately contributing to reducing immune imbalance and protecting the health status in CYP-induced immunosuppressed mice.

According to current research, GLSO contains three main bioactive components: fatty acids, steroids and triterpenoids^103^. Unsaturated fatty acids are the main and most important component of GLSO, especially including C18:1 (oleic acid), C16:0 (palmitic acid), C18:2 (linoleic acid) and C18:0 (stearic acid)^104^, which have various pharmacological effects. These unsaturated fatty acids may inhibit the production of tumor necrosis factor in the mouse macrophage preparations induced by lipopolysaccharide^105^. Besides, 14 kinds of sterols were isolated from GLSO^106^. The main sterols were directly bound to the active sites of p38 and NF-κB p65, which was likely to attenuate their phosphorylation and activation, thereby exerting anti-inflammatory activity^107^. At present, there is still a significant gap in further research on underlying immunoregulatory mechanisms behind the specific components of GLSO.

While this study provides valuable insights into GLSO role in protecting immune function, significant limitations persist. First, in the above Omics analysis, since the fold change parameter is relatively small, the present results may present false positives. Besides, the adjusted p-value could not be set as the indicator for statistical analysis due to the small sample size of the mice in this study and the biology differences, which also lacks sufficient persuasiveness. More importantly, a major limitation is the lack of further experiments to verify differential proteins and relevant pathways, which hinders the determination of GLSO mechanism in protecting immune function. Furthermore, the immune evaluation of GLSO remain gaps such as immune cell phenotypes and cytokine levels. Meanwhile, the analysis on active components of GLSO in protecting immune function also remains under-explored and warrants further investigation. Finally, the microbial function predictions and connection presented in this study between the pathways and metabolites or proteins remain speculative. The cross-talk between the microbiome, metabolome and proteome, as well as causal relationships with GLSO are also poorly understood, mainly due to the lack of long-term research. Therefore, the aforementioned deficiencies should be addressed in future research, focusing on combining the specific identified components of GLSO with the validated pathways to explore the potential immune regulatory mechanisms.

## Materials and methods

### Preparation of *G. lucidum* spore oil

GLSO was sourced from Guangzhou Hanfang Pharmaceutical Co., LTD. The spores of *Ganoderma lucidum* (Leyss.ex Fr.) Karst.) were processed using wall-breaking techniques followed by supercritical carbon dioxide extraction to obtain the oil extract. The total triterpene content in the GLSO was measured at 249 mg/g by a UV spectrophotometer, while the ergosterol content was quantified at 0.7 mg/g by HPLC.

### Animal and experimental design

Male ICR mice (5 weeks old, 18–22 g) from Beijing HFK Bioscience Co., LTD (Beijing, China), were housed under SPF-grade conditions (23 ± 2 ℃, 55 ± 5% humidity, and a 12-hour light-dark cycle) at Southwest Medical University. All animals had ad libitum access to sterilized water and feed, and the study was approved by the Committee on Use and Care of Animals of Southwest Medical University (Reference number 20190810-008; approved on 10 August 2019), and conducted in accordance with the committee’ s guidelines and relevant regulations, particularly RRIVE guidelines.

After adaptation for at least a week, the mice were randomized into four groups (*n* = 26 per group): Control (Ctrl), Model (CYP), Model + low-dose GLSO (CYP + L), and Model + high-dose GLSO (CYP + H). On days 1, 3, 5, 17, 19, and 21, the Ctrl group received intraperitoneal injections of 0.9% saline, while the other groups received 40 mg/kg CYP (MedChemExpress). Meantime, the CYP + L and CYP + H groups were also intragastrical administered 400 and 800 mg/kg GLSO (Diluted with corn oil) (10 and 20 times the effective human dose)^108,109^, respectively, while Ctrl and CYP groups were given the corresponding volume of corn oil (Longevity Flower Food Co., LTD, Shandong, China), once daily for 26 days beginning from the day of CYP injection. The body weight of the mice was measured thrice weekly (before gavage) to adjust drug dosages timely (0.1 mL/10 g body weight).

Different experimental batches included: serum hemolysin testing on Day 26 (*n* = 6 per group), fecal sample collection on Day 22 (*n* = 7–10), macrophage phagocytosis assay on Day 24 (*n* = 6), peripheral blood collection, spleen, and thymus anatomy on Day 27 (*n* = 14). Blood samples were used for leukocyte counting and serum metabolomics analysis. When the actual blood collection volume was less than 50% of the theoretical minimum effective requirement or hemolysis index was greater than 20, the serum sample was discarded. Finally, effective serum samples (*n* = 13 per group) were collected. The spleen and thymus underwent organ index calculations and quantitative proteome analysis, while the spleen also required a splenocyte activity assay. A schematic diagram of the experimental design is shown in Fig. 1A. When all mice were sacrificed by cervical dislocation, the average body weight of mice in the CON group, CYP group, CYP + L group and CYP + H group was 35.3 g, 33.65 g, 35.7 g and 35.2 g, respectively.

### Serum hemolysin assay

Mice were intraperitoneally injected with 0.2 mL of 5% fresh sheep red blood cells (SRBC, Zewell Biological Technology, Nanjing, China). Blood samples were collected from the orbital cavity five days later and centrifuged to separate the serum. The serum was then diluted 100-fold with normal saline, after which 0.5 mL of the serum was combined with 0.5 mL of 5% SRBC, 0.5 mL of 10% guinea pig serum, and 0.5 mL of normal saline. An additional reaction was performed to ascertain the 50% hemolysis value, utilizing 0.5 mL of 5% SRBC and 1.5 mL of normal saline. The mixed solutions were incubated at 37 ℃ for 30 min before being placed in ice water to terminate the reaction. Following this, the resulting supernatant was obtained by centrifugation and transferred into a 96-well plate to measure the OD (Optical density) value at 540 nm in a BioTek Synergy H1 microplate reader. The serum hemolysin content was quantified as the half hemolysis value (HC_50_), calculated using the formula: HC_50_ = (OD value of sample / OD value of 50% SRBC hemolysis) × dilution factor.

### Macrophage phagocytosis assay

Mice were weighed, which was followed by a tail vein injection of 50% India ink (100 µL/10 g BW, Solarbio, Beijing, China). Blood samples of 25 µL were collected from the inner canthus venous plexus at 3- and 11-min post-injection and respectively added to a 2 mL solution of 0.1% Na_2_CO_3_ (Sigma-Aldrich) for O.D. measurement at 600 nm in a BioTek Synergy H1 microplate reader. with the Na_2_CO_3_ solution blank. All mice were sacrificed by cervical dislocation. Then, the livers and spleens of mice were collected and weighed separately after sacrifice. The carbon clearance index (K) and phagocytosis index (α) were calculated as follows: K = (log OD _3 min_ - logOD _11 min_) /Δt; phagocytic index (α) = body weight/ (liver weight + spleen weight) × K^1/3^.

We excluded these samples on the basis of prespecified quality-exclusion criteria (e.g., incomplete organ structure, serum hemolysis index > 20). The final effective sample size was *n* = 4 per group.

### Analysis of immune organ indexes

After blood collection, the mice were sacrificed by cervical dislocation and were dissected to weigh the spleen and thymus. Organ indexes of the spleen and thymus were calculated as follows: The immune organ indexes (mg/g) = weight of the spleen or thymus (mg)/body weight (g). Tissue samples with significant accidental loss during dissection were excluded from statistical analysis, and the final number of samples for analysis was *n* = 10 per group.

### Splenic natural killer (NK) cell activity

YAC-1 cells (mouse lymphoma cell line, Pituo Biological Technology, Shanghai, China), as the target cells, were passaged 24 h before the experiment and adjusted to a concentration of 4 × 10^5^ cells/mL in RPMI 1640 medium (Thermo Fisher Scientific, U.S.). Spleens from dissected mice were ground to prepare a single-cell suspension. After centrifugation, the splenocytes were resuspended in RPMI 1640 medium, adjusting the concentration to 1 × 10^7^ cells/mL for the effector cells.

The splenocytes (2 × 10^5^ cells/well) were co-incubated with YAC-1 cells at 37 ℃ with 5% CO_2_ for 5 h in 96-well plates (200 µL/well) at a ratio of 25:1. A CCK-8 assay (Dojindo Molecular Technologies, Japan) was then performed according to the manufacturer’s instructions, with readings taken at 450 nm in a BioTek Synergy H1 microplate reader. The NK cell activity was calculated as: NK cell activity = (OD_1_-(OD_2_-OD_3_))/OD_1_, where OD_1_ represents the target cell control, OD_2_ denotes the test sample, and OD_3_ reflects the effector cell control.

### Fecal microbiome analysis

#### DNA extraction, amplification, library construction and sequencing

Fecal DNA was extracted using the CTAB/SDS method. 1% agarose gels were prepared to detect the purity of the DNA products. The distinct regions (V3-V4) of 16 S rRNA were amplified by PCR using specific primers (515 F-806R). Sequencing libraries were constructed using the Ion Plus Fragment Library kit (Thermo Scientific), and the quality of the DNA library was evaluated with a Qubit 2.0 fluorometer. The library was sequenced on the Ion S5™ XL platform, generating single-ended reads of 400 bp/600 bp.

#### Sequencing data processing

Sample data were separated from the reads based on unique Barcodes, and the raw reads were obtained by truncating the barcode and primer sequences. Quality filtering of the raw reads was performed using the Cutadapt (V1.9.1, http://cutadapt.readthedocs.io/en/stable/) as a quality-controlled procedure to obtain high-quality clean reads. These reads were compared with the reference database (SILVA database, https://www.arb-silva.de/) using the UCHIME algorithm (http://www.drive5.com/usearch/manual/uchime_algo.html) to identify and eliminate chimeric sequences, resulting in high-quality clean reads.

#### OTU cluster and species annotation

Sequence analysis was performed using Uparse software (Uparse v7.0.1001, http://drive5.com/uparse/), clustering sequences with 97% similarity or higher into identical OTUs (Operational Taxonomic Units). For further annotation and species analysis, representative sequences for each OTU were selected via the Mothur algorithm and the SSUrRNA database (http://www.arb-silva.de/) with a threshold of 0.8-1. enabling taxonomic information retrieval and community composition analysis across various taxonomic levels. Multiple sequence alignments were conducted using MUSCLE software (Version 3.8.31, http://www.drive5.com/muscle/) to explore phylogenetic relationships among all OTUs. The OTU abundance data were normalized based on a standard sequence number corresponding to the sample with the fewest sequences.

#### Microbial diversity analysis and function prediction

Alpha and beta diversity were assessed using QIIME (Version 1.7.0) and visualized with R software (Version 2.15.3) with Wilcoxon rank-sum test. The data structure was analyzed using non-metric multidimensional scaling (NMDS) based on Bray-Curtis dissimilarity matrices. Functional predictions, derived from the KEGG database, were conducted according to the 16 S sequencing data (PERM ANOVA p-value < 0.05).

### Serum metabolomics analysis

#### Metabolites extraction

Mix 100 µL of serum with 400 µL of pre-chilled 80% methanol and vortexed for 1 min. Incubate at -80 ℃ for 2 h to precipitate the protein and centrifuge. Transfer the supernatant to a fresh Eppendorf tube with a 0.22 μm filter and centrifuge again. Store the samples at -80 °C until UHPLC-MS/MS analysis. For LC-MS system conditioning and quality control, mix equal volumes of all samples as a QC sample.

#### UHPLC-MS/MS analysis

UHPLC-MS/MS analysis was performed on a Q Exactive mass spectrometer (Thermo Fisher) with a Vanquish UHPLC system. Sample separation was performed on the Hypersil Gold column (100 × 2.1 mm, 1.9 μm) at a flow rate of 0.2 mL/min, with the column temperature at 40 ℃. The eluents for the positive polarity mode were eluent A (0.1% formic acid in water) and eluent B (methanol). The eluents for the negative polarity mode were eluent A (5 mM ammonium acetate, pH 9.0) and B (methanol). The elution gradient was set as follows: 2%B, 0–1.5 min; 2-100%B, 1.5–12.0 min; 100% B, 12.0–14.0 min; 100-2% B, 14.0–14.1 min; 2% B, 14.1–16 min. MS assays were performed in positive and negative modes with a spray voltage of 3.2 kV, capillary temperature of 320 °C, sheath gas flow rate of 35 arb, auxiliary gas flow rate of 10 arb, and injection volume of 10 µL.

#### Metabolome data processing

Raw MS data files were converted into mzXML format and then processed for peak picking, peak grouping, retention time correlation, and second peak grouping using the XCMS (version 3.9.3) package. The main parameters were set as follows: retention time tolerance, 0.2 min; actual mass tolerance, 5ppm; signal intensity tolerance, 30%; signal/noise ratio, 3; and minimum intensity, 100,000. MetaX (version 1.14.19) package was used for statistics analysis. Metabolite features detected in less than 50% QC samples or 80% of biological samples were removed, and the remaining peaks with missing values were imputed with the k-nearest neighbor algorithm and normalized using the probabilistic quotient normalization. Next, low quantitative quality features with a relative standard deviation higher than 50% were eliminated. Metabolites were then identified with their fragment spectra matching against several MS/MS databases (HMDB, Massbank, Lipidblast, and an in-house database) using MSDIAL software (version 2.94). Mass tolerance was set to 0.01 for MS^1^ and 0.05 for MS/MS, with an identification score cut-off of 0.7. Cytoscape was used to establish the metabolite and pathway network with hypergeometric test p-value < 0.05. Scatter plot of VIP from PLSDA and p-value from Kruskal-Wallis test for significantly changed metabolite features with FC > 1.2, VIP > 1 and p-value < 0.05.

### Thymus proteomics analysis

#### Total protein extraction

The sample was ground in liquid nitrogen and lysed using a lysis buffer of 100 mM ammonium bicarbonate (pH 8), 6 M urea, and 0.2% sodium dodecyl sulfate. Following the lysis process, ultrasonication was performed on ice for 5 min. The lysate was centrifuged, and the supernatant was transferred to a clean tube. The extracts were reduced with 10 mM dithiothreitol for 1 h at 56 ℃, followed by alkylation with iodoacetamide for 1 h at room temperature in the dark. The samples were mixed with 4 volumes of pre-cooled acetone and incubated at -20 ℃ for at least 2 h. After centrifugation, the pellet was washed with cold acetone and dissolved in a buffer containing 0.1 M triethylammonium bicarbonate (pH 8.5) and 6 M urea.

#### TMT labeling of peptides

Total protein was quantified with the Bradford method and 120 µg proteins were digested with 3 µg trypsin at 37 °C overnight. The peptides were enriched using a C18 column and labeled with Tandem Mass Tag (TMT) reagent according to the manufacturer’s protocol. Labeled peptide mixtures were then pooled, and generated 10 fractions using a High pH reversed-phase C18 column (Waters BEH C18, 250 × 4.6 mm, 5 μm) linked to a Rigol L3000 HPLC system. All fractions were dried under vacuum and kept at -80 °C until the UHPLC-MS/MS analysis.

#### UHPLC-MS/MS analysis

Each fraction was injected into a home-made analytical column (15 cm×150 μm, 1.9 μm), using a 90 min linear gradient elution. The separated peptides were analyzed by Q Exactive HF-X mass spectrometer (Thermo Fisher Scientific). Full scan ranges from m/z 350 to 1500 with resolution of 60,000 (at m/z 200), an automatic gain control (AGC) target value was 3 × 10^6^ and a maximum ion injection time was 20 ms. The top 40 precursors of the highest abundant in the full scan were selected and fragmented by higher energy collisional dissociation (HCD) and analyzed in MS/MS, where resolution was 45,000 (at m/z 200), the AGC target value was 5 × 10^4^ the maximum ion injection time was 45 ms, a normalized collision energy was set as 32%, an intensity threshold was 1.9 × 10^5^, and the dynamic exclusion parameter was 20 s.

#### Proteomics data processing

The MS/MS data were analyzed against the UniProt *Mus musculus* reference (85,188 sequences downloaded in 2019.01.18) using Proteome Discoverer 2.2 (Thermo Fisher Scientific). The analysis was conducted with the following parameters: a mass tolerance of 10 ppm for precursor ions and 0.02 Da for product ions. Carbamidomethylation was defined as a fixed modification, while oxidation of methionine (M), acetylation of the N-terminus, and TMT 10-plex of tyrosine and lysine were designated as variable modifications. A maximum of 2 missed cleavage sites was allowed. The identified proteins contained at least 1 unique peptide with a false discovery rate of less than 1%. Proteins with similar peptides that could not be distinguished by MS/MS analysis were classified as the same protein group. Reporter Quantification (TMT 10-plex) was used for TMT quantification.

### Statistical analysis

Statistical significance tests, including the Wilcoxon rank-sum test, one-way analysis of variance (ANOVA), Kruskal-Wallis rank-sum test, hypergeometric test, and Spearman correlation were performed using R software (version 4.1.0; https://www.r-project.org/). Partial least squares discriminant analysis (PLS-DA) was performed with a RoPLS package. Permutational multivariate analysis of variance (PERMANOVA) was performed with a vegan package. The differential molecules were illustrated with a heatmap by using the R software (version 4.1.0; https://www.r-project.org/) with the ComplexHeatmap package (version 2.26.1; http://bioconductor.org/packages/release/bioc/html/ComplexHeatmap.html)^110^. KEGG database was used for omics function analysis^111–113^.

## Conclusion

The present study elucidated the characteristics of alterations mediated by GLSO among gut microbiota, metabolomics, and proteomics about immunity protection in immunosuppressed mice. A high dose of 800 mg/kg GLSO administration was shown to significantly ameliorate impaired innate and adaptive immune functions in immunosuppressed mice. Comprehensive microbiome, metabolomic, and proteomic analyses revealed that the GLSO initiated the structural rearrangement in gut microflora and the enhancement of microbial diversity with potentially an increase in healthy microorganisms and a decrease in pathogenic species, accompanied by notable alterations in metabolite profiles and protein expression. Furthermore, GLSO treatment alleviated immune dysfunction in CYP-induced immunosuppressed mice. Notably, 5 metabolites (propionic acid, beta-glycyrrhetinic acid, 3-aminosalicylic acid, creatine, and 2-phenylacetamide) and 5 proteins (Slc9a9, Blm, Hk3, AP1M2, and J chain) might be assumed as pivotal effectors in the GLSO-mediated protection of immune functions. The distinctive alterations and relevant pathways identified in the microbes-metabolite-proteins axis might play a crucial role in the immune protection process facilitated by GLSO. The findings might provide significant insights into the influence of GLSO on the protection of immune functions, paving the way for further research in this area.

## Supplementary Information

Below is the link to the electronic supplementary material.

Supplementary Material 1

Supplementary Material 2

Supplementary Material 3

## Data Availability

The Proteome datasets generated and/or analysed during the current study are available in the PRIDE repository (ProteomeXchange accession: PXD067907; Project Webpage: https://www.ebi.ac.uk/pride/archive/projects/PXD067907). The raw sequence data of Metagenome reported in this paper have been deposited in the Genome Sequence Archive (Genomics, Proteomics & Bioinformatics 2025) in National Genomics Data Center (Nucleic Acids Res 2025)^114,115^, China National Center for Bioinformation / Beijing Institute of Genomics, Chinese Academy of Sciences (GSA: CRA038543) that are publicly accessible at https://ngdc.cncb.ac.cn/gsa/browse/CRA038543. The data of Metabolome reported in this paper have been deposited in the OMIX, China National Center for Bioinformation / Beijing Institute of Genomics, Chinese Academy of Sciences (https://ngdc.cncb.ac.cn/omix: accession no.OMIX014930).

## Abbreviations

AGC: An automatic gain control
ANOVA: One-way analysis of variance
BW: Body weigh
Ctrl: Control
CYP: Cyclophosphamide
CYP + L: Cyclophosphamide + low-dose *Ganoderma lucidum* spore oil
CYP + H: Cyclophosphamide + high-dose *Ganoderma lucidum* spore oil
GLSO: *Ganoderma lucidum* spore oil
GPCRs: G protein-coupled receptors
HC_50_: Half hemolysis value
HCD: Higher energy collisional dissociation
HPLC: High Performance Liquid Chromatography
NETs: Neutrophil extracellular traps
NK: Natural killer
NMDS: Non-metric multidimensional scaling
OD: Optical density
OTUs: Operational Taxonomic Units
PERMANOVA: Permutational multivariate analysis of variance
PLS-DA: Partial least squares discriminant analysis
SCFAs: Short-chain fatty acids
SRBC: Sheep red blood cells
TMT: Tandem Mass Tag
UHPLC-MS/MS: Ultra-high-performance liquid chromatography coupled with tandem mass spectrometry
YAC-1 cells: Mouse lymphoma cell line
